# Variability in Cold Tolerance of Food and Clinical *Listeria monocytogenes* Isolates

**DOI:** 10.3390/microorganisms11010065

**Published:** 2022-12-26

**Authors:** Peter Myintzaw, Vincenzo Pennone, Olivia McAuliffe, Máire Begley, Michael Callanan

**Affiliations:** 1 Department of Biological Sciences, Munster Technological University, Bishopstown, T12 P928 Cork, Ireland; 2 Teagasc Food Research Centre, Moorepark, Fermoy, Co., P61 C996 Cork, Ireland

**Keywords:** *Listeria*, cold tolerance, growth rate, food matrices, typing

## Abstract

The aim of this study was to investigate the level of strain variability amongst food and clinical *Listeria monocytogenes* isolates growing at low temperatures (4 and 7 °C) in both laboratory media and real food matrices. Isolates (*n* = 150) grown in laboratory media demonstrated a large variation in growth profiles measured using optical density. Overall, it was noted that clinical isolates exhibited a significantly higher growth rate (*p* ≤ 0.05) at 7 °C than the other isolates. Analysis of variance (ANOVA) tests of isolates grouped using Multi Locus Sequence Typing (MLST) revealed that clonal complex 18 (CC18) isolates were significantly (*p* ≤ 0.05) faster growing at 4 °C than other CC-type isolates while CC101, CC18, CC8, CC37 and CC14 were faster growing than other CC types at 7 °C. Euclidean distance and Ward method-based hierarchical clustering of mean growth rates classified 33.33% of isolates as faster growing. Fast and slow growing representative isolates were selected from the cluster analysis and growth rates were determined using plate count data in laboratory media and model food matrices. In agreement with the optical density experiments, CC18 isolates were faster and CC121 isolates were slower than other CC types in laboratory media, UHT milk and fish pie. The same trend was observed in chocolate milk but the differences were not statistically significant. Moreover, pan-genome analysis (Scoary) of isolate genome sequences only identified six genes of unknown function associated with increased cold tolerance while failing to identify any known cold tolerance genes. Overall, an association that was consistent in laboratory media and real food matrices was demonstrated between isolate CC type and increased cold tolerance.

## 1. Introduction

The capabilities of the psychrotrophic, facultatively anaerobic Gram-positive bacterium *L. monocytogenes* to proliferate at refrigeration temperature remains a significant food safety concern and a public health issue. According to a recent European Food Safety Authority (EFSA) report, 1876 human cases of listeriosis (0.42 cases per 100,000 persons) were reported in 2020, with a high fatality rate of 13.0% [1]. This makes listeriosis one of the most serious foodborne diseases under EU surveillance. In the United States, 928 cases of listeriosis (0.28 cases per 100,000 persons) were reported in 2019 [2].

To distinguish isolates, *L. monocytogenes* is currently subtyped into thirteen serotypes, four genetic lineages and multiple clonal complexes. The subtyping plays a major role in application of more rapid, precise and efficient foodborne disease surveillance, outbreak detection and source tracking throughout the food chain [3]. Amongst the many subtyping methods, multi-locus sequence typing (MLST) based on seven housekeeping genes has high discriminatory power, allowing grouping of isolates into clonal complex (CC) types [4]. In silico whole-genome sequence (WGS) data are also used for species confirmation and sequence type or strain type (ST) determination [5,6,7]. In addition, pan-genomic analysis using software such as Roary allows core-pan genome construction based on sequence similarity [8] and has been applied to WGS data as an effective approach to understand genetic diversity as well as to gain insights into specific microbial clades. Furthermore, Scoary’s [9] correlations of genes sorted by trait strength to pan-genome components have potential for understanding genetic determinants of particular phenotypes.

As *L. monocytogenes* is mainly transmitted through contaminated food and the organism is able to grow at low temperatures, refrigerated ready-to-eat (RTE) food products with longer shelf life are most at risk. Codex Alimentarius Commission CAC/GL 61-2007 and European commission EC No 2073/2005 require that RTE foods not exceed the limit of 100 CFU/g for *L. monocytogenes* at any point during their shelf life [10,11]. Typically, consumers purchase food products and store them in their refrigerators, intending to use them within a few days. However, not all household refrigerators are able to maintain the recommended temperature of 5 °C. According to a French survey, the average refrigerator temperature is 6.6 °C, with a minimum of 0.9 °C and a maximum of 11.4 °C [12]. *L. monocytogenes* can grow at temperatures as low as −0.4 °C [13], which allows the organism to grow to infectious doses even at low temperatures and consequently infect high-risk individuals.

With regard to food safety and public health, it is important to understand at the individual strain level the variability and molecular mechanisms which allow growth in chilled distribution chains. *L. monocytogenes* encounters a multitude of cold stress challenges such as blast chilling, blast freezing and thawing along the food chain, which the organism must efficiently adapt to in order to survive and proliferate [14]. The molecular mechanisms that regulate and adapt *L. monocytogenes* to multiple stresses in the food chain have been studied in detail and linked to the presence of specific genes. Some genes are part of the general stress response while others are specific to the individual stress. Experimental evidence has identified genes involved in cold adaption by mutagenesis, genes expression and transcriptional analysis using reference strains. In addition, the availability of whole genome sequences from multiple isolates can now provide detailed insights into gene distribution and the link with strain variability.

The value of whole genomic information in the study of the stress responses of *L. monocytogenes* has already been reported for organic acids [6] and low temperature growth [7,15]. Specifically, variation in the maximum growth rates of large numbers of *L. monocytogenes* strains at 4 °C and 2 °C has previously been examined [7,15], and genome-wide associations have been explored based on core genome single nucleotide variants [7] and at pan-genome scale [15]. Here we report on variability in the growth rates of a bank (*n* = 150) of *L. monocytogenes* isolates sourced specifically in Ireland growing at chill (4 °C) and mild abuse (7 °C) temperatures encountered along the food chain and referenced in the EURL *Lm* Technical guidance [16]. The aim of the study was to gain a better understanding of the variability of *L. monocytogenes* growth at realistic refrigeration temperatures in laboratory media and real food matrices.

## 2. Materials and Methods

### 2.1. Strains and Culture Condition

A total of 150 *L. monocytogenes* isolates from various food, food production environment and clinical sources were obtained from the *Listeria* collection at Teagasc Food Research Centre, Moorepark, Co Cork. We have previously reported the source information and whole genome sequencing data of the isolates [6]. Individual isolates were stored at −80 °C (long-term) and −20 °C (short-term) in 40% (*v*/*v*) glycerol and were resuscitated in 10 mL Brain Heart Infusion broth (BHIB, Merck, Ireland) and incubated at 37 °C for 24 h. Subsequently, cultures were streaked onto BHI agar plates and the plates were incubated for 48 h at 37 °C. BHI agar plates containing streaked isolates were stored at 4 °C for short-term storage. The starting inoculum for each isolate was prepared from a single colony and inoculated into 10 mL BHI broth for 18 h at 37 °C. Details of selected food matrices purchased from local supermarket in Cork city are listed in Table 1.

### 2.2. Growth Rate Determination by Optical Density (OD) Measurement

Overnight (18 h) cultures were diluted (1:10,000) ≈ 1 × 10^5^ CFU/mL in saline solution (NaCl 0.85%) and 2 µL of each diluted culture was aliquoted in a well of a 96 well plate with 198 µL BHIB in triplicate (technical replicates). Total plate counting was employed to determine the inoculum concentration. For each 96 well plate, reference strains *L. monocytogenes* EGD-e and F2365 acted as positive controls and 200 µL sterile BHIB without culture were incubated alongside as a negative control. Each test plate was sealed with plate sealer Thermo Scientific™ Microtiter™ Dublin, Ireland and incubated at 4 and 7 °C, respectively, in a domestic fridge (Whirlpool ARC 104/1 121 Litre A+ Under counter Fridge, UK for 7 °C and Khol mini 50 L tabletop fridge, UK for 4 °C). Temperature fluctuation was monitored during incubation using temperature data logger (EasyLog Lascar electronic, Wiltshire, UK). At each time point microtiter plates were taken out from the fridge, the seals were swiftly replaced aseptically with fresh ones and OD_600_ readings were taken following 30-sec maximum intensity shaking from T0 (0 h) to T400 using a microplate reader (Multiskan Sky, Agilent, Waldbronn, Germany). Plates were outside the fridge for approximately 60 s.

### 2.3. Estimation of Growth Rates and Fast or Slow Growth Clustering

Raw data were gathered in Microsoft Office Excel 2013 and growth modelling and growth curves were visualised with the statistical software R 4.0.1 Growthcurver package [17,18]. The estimation of the growth rate (OD_600_ µ h^−1^) of *L. monocytogenes* isolates was calculated by fitting the modified Gompertz model available at https://padpadpadpad.github.io/post/calculating-microbial-growth-rates-from-od-using-rolling-regression/ accessed on 14 December 2021. Growth rate data obtained from 4 and 7 °C were examined in R version 4.1.1. and grouped using Euclidean distance and Ward clustering.

### 2.4. Genotyping and Pan-Genome Analysis

Information relating to in silico Multi Locus Sequence Typing (MLST), lineage determination, clonal complex (CC) assignment, the presence of plasmids and the presence of virulence genes for all 150 isolates has previously been published by our group [6]. The whole-genome annotation was carried out using the Prokka pipeline at Use Galaxy web interface (https://usegalaxy.org/ accessed on 24 October 2021) [19,20] with the default parameters. Prokka output GFF3-files were used to extract the pan-genome of the 150 *L. monocytogenes* isolates with Roary version 3.13.2 [8] available at (http://sanger-pathogens.github.io/Roary/ accessed on 04 January 2022). The presence or absence of gene files generated by Roary was coupled with an observed cold growth trait in Scoary v1.6.16 available at (https://github.com/AdmiralenOla/Scoary accessed on 21 January 2022), (Gene-wise counting and Fisher’s exact tests for trait) to establish gene clusters associated with each trait [9]. The Roary and Scoary output files coupled with phenotype data were visualised using the online interactive tool phandango [21]. *L. monocytogenes* gene sequences previously shown to be linked to cold tolerance were identified as described previously [6]. Briefly, gene sequences were collected from Listilist (http://genolist.pasteur.fr/ accessed on 20 March 2021) and are listed in Supplementary Table S1. The presence or absence of genes in each genome was established by performing a standalone local NCBI BLAST+ 2.10.0+ executable with a cutoff of >95% nucleotide identity and an e-value of <10^6^ (https://ftp.ncbi.nlm.nih.gov/blast/executables/blast+/LATEST/ accessed on 2 April 2021).

### 2.5. Measurement of Microbial Growth by Plate Counts in Laboratory Media and Food Matrices

Measurement of the growth of selected isolates based on their CC-type (Table 2) that exhibited significantly faster or slower growth as determined by OD measurements was examined in BHIB and three different food matrices. In addition, the serotype 4b strain, *L. monocytogenes* F2365, was added as a reference strain. UHT treated milk, chocolate milk and fish pie (baby food) were purchased as commercially sterile products, and to confirm sterility 1 g of each was diluted in 9 mL of 0.85% NaCl (LabM, Lancashire, UK) solution and 100 µL aliquots were spread-plated on BHI agar. The plates were inspected for evidence of microbial growth after 48 h of incubation at 30 °C. Starting *L. monocytogenes* inocula were prepared from diluted 18 h culture with saline solution (NaCl 0.85%) to a concentration of approx. 1000 CFU/mL. 100 µL and 400 µL of the inoculum were then inoculated into 9.9 mL and 39.6 g of BHI broth and food matrices, respectively. Each test unit was incubated at 4 and 7 °C. Following inoculation, the test units were vortexed, mixed thoroughly and enumerated immediately to provide a T0 reading. At each time point, 1 g of food samples and 1 mL of liquid samples were serially diluted in saline solution and 100 µL of each dilution was spread-plated onto BHI agar for enumeration. The spot-plating technique where 10 µL of each dilution was spotted on BHI agar plates was employed as bacterial numbers increased [22]. After 48 h of incubation at 30 °C, colonies were manually counted. For each isolate and each temperature condition, cold growth experiments were performed using three independent biological replicates and a maximum of 679 h. Growth rates were calculated using DMFit version 3.5 in Microsoft excel provided by ComBase (available online: https://www.combase.cc/DMFit_Excel.aspx, accessed on 29 November 2022).

### 2.6. Data Analysis

Statistical analyses were performed with R version 4.0.1. A value of *p* ≤ 0.05 was considered statistically significant in each test. The distributions of data were examined for a normality test and homogeneity of variance. Means and standard deviations (SDs) were calculated. A one-way ANOVA test with Bonferroni correction analysis was employed to compare the mean scores between groups. A chi-square test of independence or Fisher’s exact tests (when expected value was less than five) were employed to investigate the presence of genes previously shown to be linked with cold tolerance in WGS and isolates with a fast growth phenotype.

## 3. Results

### 3.1. Cold Tolerance of Isolates

**Figure 1:**
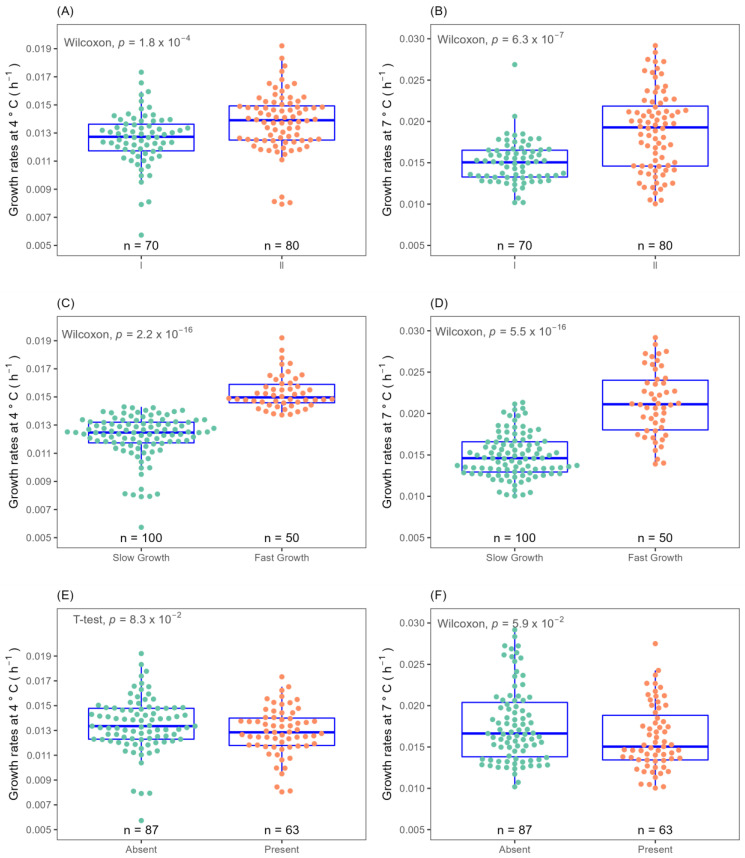
Isolates clustered by optical density determined growth rates at 4 and 7 °C according to lineage I (green); lineage II (orange) (**A**,**B**), hierarchical cluster slow growth (green); fast growth (orange) (**C**,**D**) and absence of plasmid (green); presence of plasmid (orange) (**E**,**F**).

The growth of food and clinical *L. monocytogenes* isolates (*n* = 150) was monitored in laboratory media (BHIB) at temperatures of 4 and 7 °C by measuring optical density. OD_600_ measurements were fitted with multiple growth models including Baranyi, logistic, Gompertz, modified-Gompertz, Richards and model-free splines models to determine a suitable method for growth rate calculation. The result that the modified-Gompertz model gave was the closest rate to the prediction made by the ComBase online tool with the same parameters. A heterogeneous cold stress growth response was observed across the 150 isolates studied. Overall, growth rate µ and lag time λ calculated by the modified-Gompertz model ranged from 0.006–0.019 with $\bar{x}$ of 0.013 for 4 °C, 0.010–0.031 with $\bar{x}$ of 0.017 for 7 °C, λ 25.662–294.400 with $\bar{x}$ of 72.860 for 4 °C and λ 12.908–141.693 with $\bar{x}$ 54.320 for 7 °C. See Supplementary Table S2 for detail. Based on dendrogram visualisation of Euclidean and Ward hierarchical clustering, isolates were grouped at both temperatures into two clusters, fast (*n* = 50 or 33.33% of strains) and slow (*n* = 100) growing (Figure 1C,D). 

### 3.2. Pan-Genome Analysis

**Figure 2:**
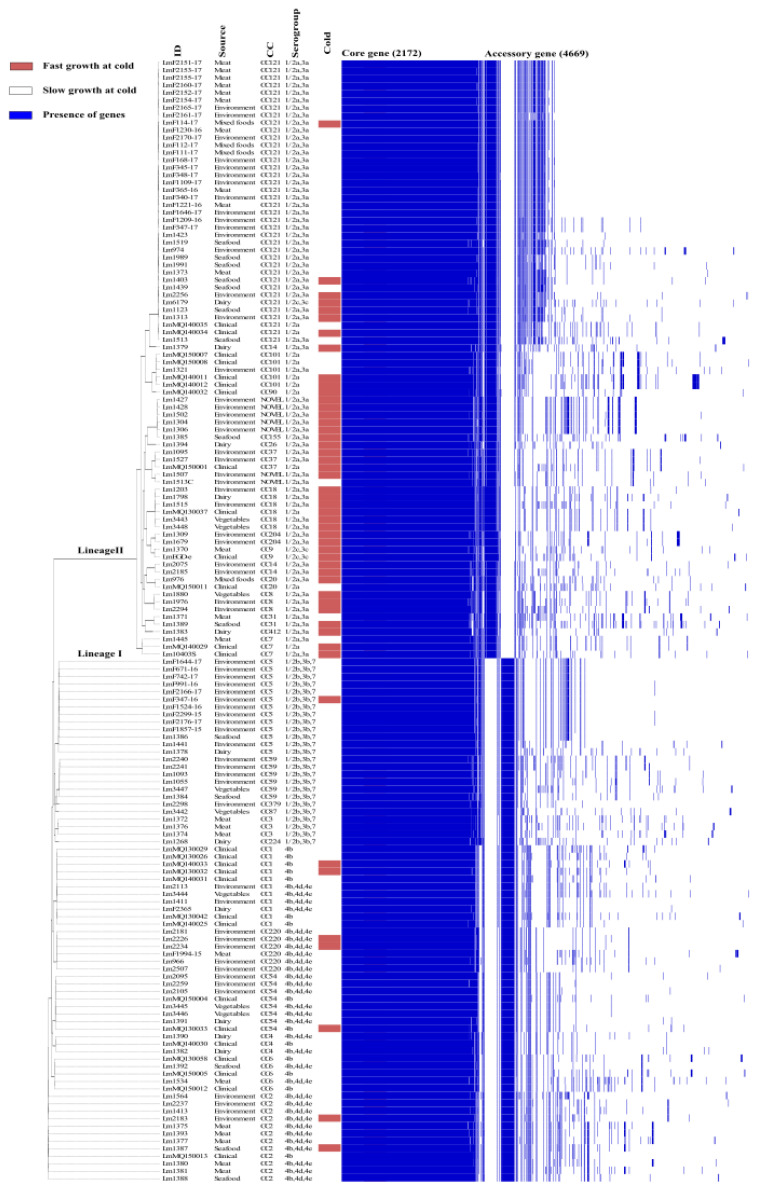
Alignment of MLST classification of 150 *L. monocytogenes* isolates with a pan-genome matrix of core and accessory genes (presence indicated by blue bars). Isolate cold growth profile (cold) is also indicated by red (fast) and purple (white) bars along with the source of the isolates.

Genes belonging to the accessory *Listeria* genome are frequently involved in various stress tolerances and have a significant impact on the ability to persist in a particular environment [23,24]. Therefore, the pan-genome was analysed to determine the genomic diversity among all *L. monocytogenes* isolates. The pan genome of the 150 isolates (Figure 2) contains a total of 6841 genes, including 2172 core genes (found in 99 percent of isolates) and 4669 accessory genes. Recent studies reported similar numbers of core genes, 2014 genes in 51 isolates [15] and 2354 genes in 11 strains [25]. In addition, a total of 798 unique genes were identified with an average of five isolate specific genes among the accessory genes.

### 3.3. Association of Cold Growth Phenotypes with Isolate Subtypes and Stress Genes

**Figure 3:**
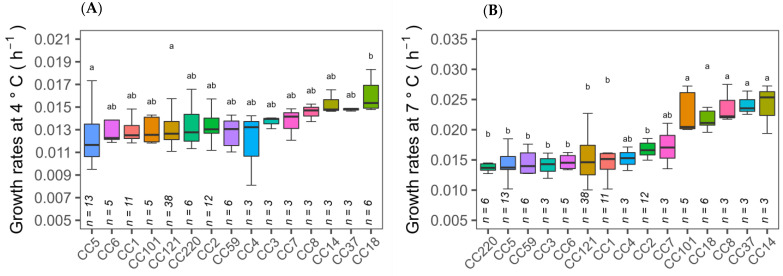
Isolates clustered by optical density determined growth rates at 4 and 7 °C according to CC type (**A**,**B**). Error bars represent standard deviations of three or more isolates’ growth rate within respective CC type. Clonal complex type with different denoted letters within the same stress are significantly different (*p* < 0.05).

The 150 isolates consist of a diverse number of CC types, as determined by MLST typing according to the Pasteur Institute classification scheme [6]. There are twenty eight different CC types in total amongst the 150 isolates of which seven CC types are novel. Analysis of variance (ANOVA) tests on the ability of CC types to grow at cold temperature revealed that CC18 isolates had significantly (*p* ≤ 0.05) higher growth rates at 4 °C than other CC types, while CC101, CC18, CC8, CC37 and CC14 isolates had higher cold tolerance at 7 °C (Figure 3A,B). Therefore, specific cold tolerance was associated with isolates grouped by their CC types. A similar trend of faster growth of *L. monocytogenes* CC18 and CC14 strains and slower growth of CC1 compared to other CC types was observed at 2 °C using visual turbidity inspection [15]. One further recent study reported that strain 2MOB079LM, which belongs to CC14, grew significantly better than strain 1513COB874 belonging to CC121 at 7.8 °C in BHIB [26]. The strain variation in growth rates at cold temperatures was shown to be significantly different among CC types in the same study. MLST typing sub divided isolates into lineage l and ll. Lineage II isolates were statistically faster growing than lineage I at both 4 and 7 °C (Figure 1A,B). The presence or absence of plasmid as detected by the CGE server had no effect on the ability to grow at both cold temperatures (Figure 1E,F). Alignment of the MLST-based phylogenetic tree of all 150 *L. monocytogenes* isolates with cold stress profile, source and genome construct is shown in Figure 2. This figure highlights the Lineage 1 and II genome conservation and that the majority of the fast-growing isolates identified by Euclidean and Ward analysis are clustered around specific branches and demonstrate some genome conservation. However, Scoary’s correlations of genes sorted by trait strength [9] revealed only six potential genes linked to faster growth in cold conditions and all of these were unknown hypothetical genes or experimentally uncharacterised genes. Table 3 displays the six genes with predicted function based on BLASTX alignment at NCBI (https://blast.ncbi.nlm.nih.gov/Blast.cgi, accessed on 29 November 2022).

**Figure 4:**
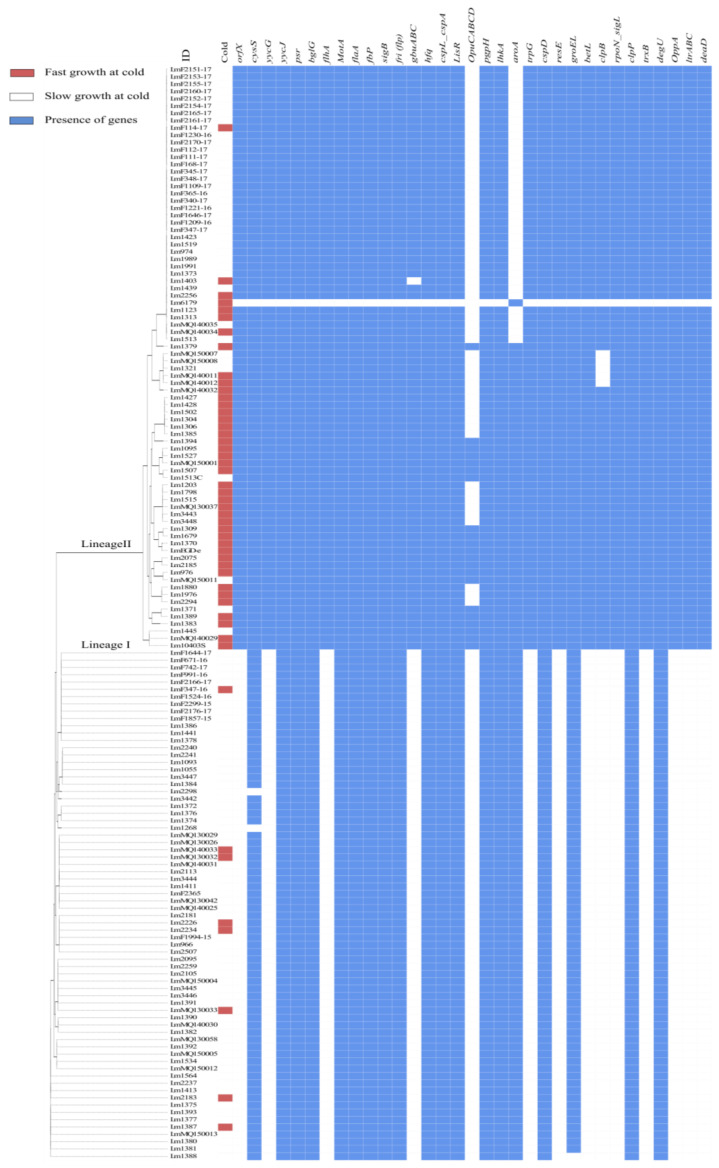
Alignment of MLST classification of *L. monocytogenes* isolates and BLAST determined the presence (cutoff > 95% nucleotide identity) of previously identified stress tolerance loci in *L. monocytogenes* in red (presence) or white (absence). The cold temperature growth profile of the isolates is also indicated with orange (fast) or purple (slow) bars.

Gene associations with faster growth at low temperatures were also investigated using standalone BLAST with a list of genes previously linked to cold tolerance in *L. monocytogenes* by various laboratory experiments (see Supplementary Table S1). A chi-square test of independence or Fisher’s exact test revealed that the presence of *orfX*, *yycG*, *flhA*, *gbu*-A;B;C, *OpuC*-A;B;C;D, *aroA*, *trpG*, *resE*, *betL*, *clpB*, *rpoN*_*sigL*, *trxB*, *OppA* and *deaD* were significantly (*p* < 0.05) associated with faster growth at a low temperature (Figure 4).

### 3.4. Growth Rates Determined by Standard Plate Count in Media and Food Matrices

**Figure 5:**
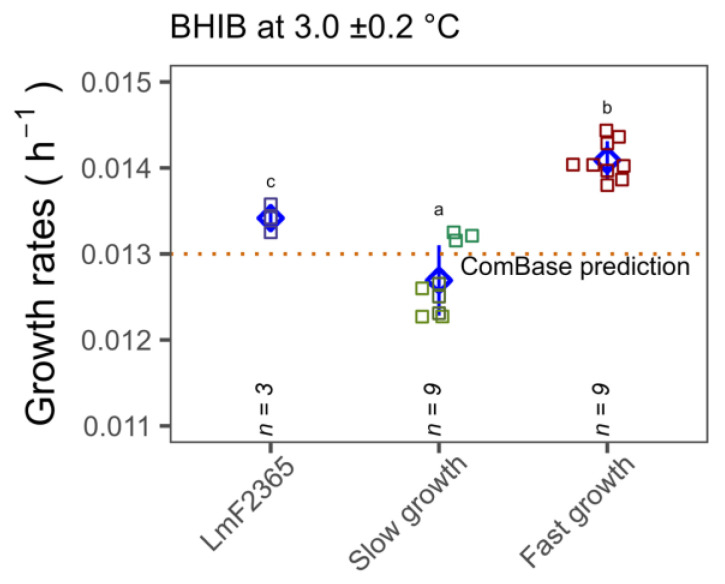

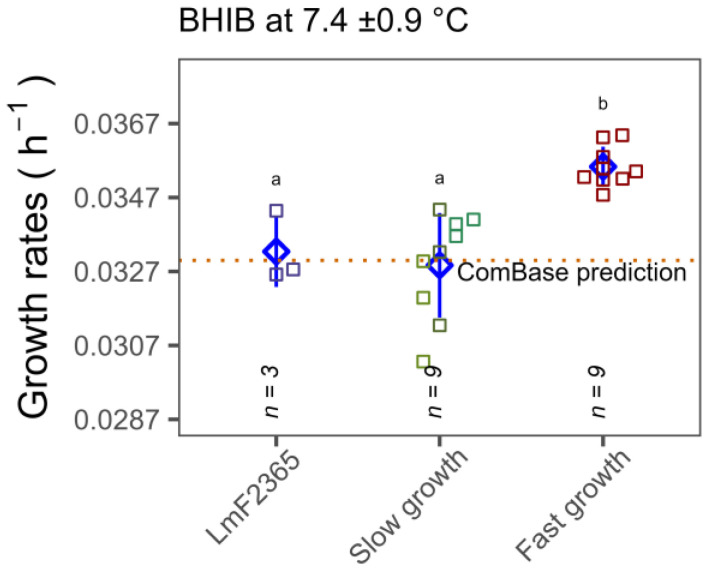
Comparison of the growth rates of seven selected isolates (slower growing in green Lm1372, Lm1378 and Lm1991 (CC3, CC5 and CC121, respectively) and faster growing in red Lm1515, Lm3443, Lm3448 (CC18)) and reference strain in light blue, LmF2365 (CC1), at 3 ±0.2 and 7.4 ± 0.9 °C in BHIB. Error bars (pointrange in blue) represent standard deviations of three independent assays for each isolate. Different lower case letters indicate significant differences at *p* < 0.05.

As the OD growth rate experiments highlighted significant variation in cold temperature tolerance among isolates, further growth rate experiments were carried out at low temperatures by performing standard plate counts with specific CC type isolates that exhibited significantly faster and slower growth. Temperature logger data revealed that mean temperatures during the growth experiments were 3.0 ± 0.2 and 7.4 ± 0.9, respectively. In agreement with spectrophotometric data, it was observed that the growth rates of CC18 isolates at 3 and 7.4 °C (0.014 ± 0.0002 and 0.0356 ± 0.0005 h^−1^) was significantly higher than CC3, CC5 and CC121 isolates (0.013 ± 0.0004 and 0.033 ± 0.001 h^−1^) (*p* < 0.05) (Figure 5). It is worth noting that the growth rate of the reference strain F2365 was comparable with the ComBase prediction for the same conditions. The growth rates of the slower growth CC types, namely CC3, CC5, CC121 isolates, were slightly lower than ComBase predictions but the CC18 group showed significantly faster growth than ComBase at both temperatures.

**Figure 6:**
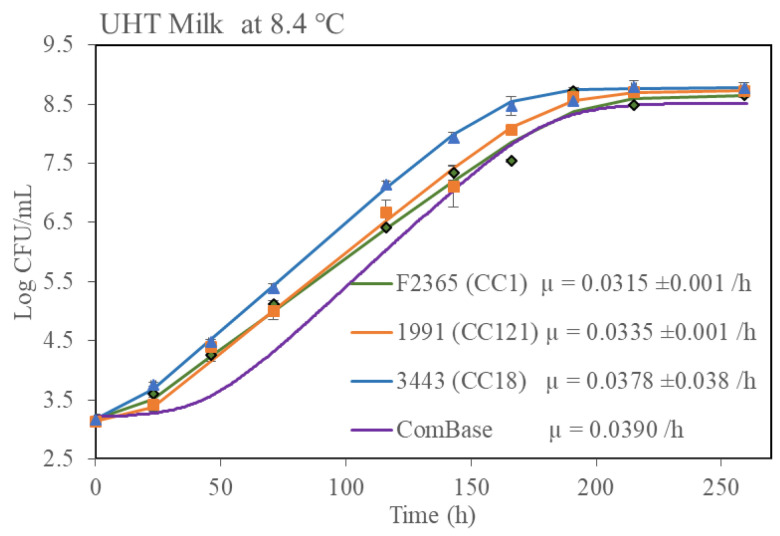

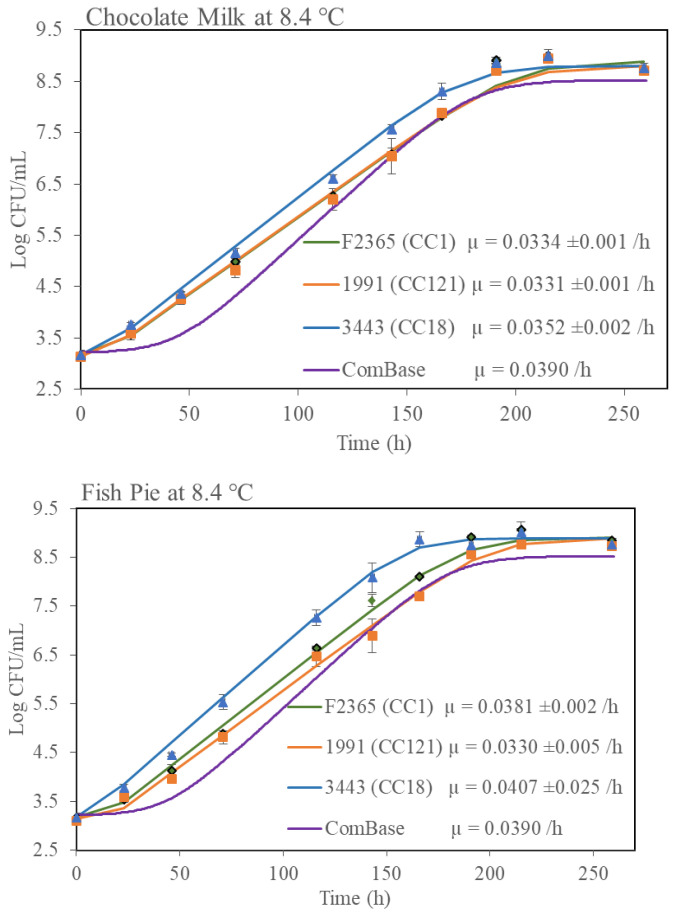
Growth kinetics of three *L. monocytogenes* isolates in three different food matrices at 8.4 °C. Growth rates (µ) shown were calculated by using DMFit excel version 3.5 provided by ComBase. Error bar represented standard deviation of biological repeats at respective time point.

Since it has been shown that food microstructure can have a major impact on microbial growth kinetics [27], it was decided to perform further growth rate studies with one selected isolate from each group (fast growth and slow growth) in three real food matrices. Risk- and evidence-based screening were performed in the Rapid Alert System for Food and Feed (RASFF) food notification tool regarding *L. monocytogenes* contamination in food for human consumption between September 2020-March 2022 in the EU. From the resultant data, it was determined that fish and fish products and dairy products were impacted most by *L. monocytogenes* contamination (Supplementary Figure S1). Therefore, growth data in milk, chocolate milk and a fish pie product was generated (Figure 6). Growth of the strains in the UHT milk and fish pie was similar to the growth observed in BHIB in that isolate Lm3443 (the fast growth CC18 representative) grew the fastest and isolate Lm1991 (the slow growth CC121 representative) grew slower. A similar trend was observed in the chocolate milk but the differences were not statistically significant. It was also noted that all CC1 and CC18 isolates grew better in the fish pie than in the dairy products.

## 4. Discussion

The ability of *L. monocytogenes* isolates (*n* = 150) from multiple sources to grow in cold temperatures was explored. In silico MLST typing suggested that the isolates in this study contain a wide diversity of CC types, with a total of twenty eight. Variation in growth rates has been studied previously with 166 isolates of thirty CC types at 4 °C [7] and 51 isolates belonging to seventeen CC-types at 2 °C [15]. This complementary study with 150 isolates contained different groups of CC types and growth rates were analysed at two different low temperatures, 4 and 7 °C. Analysis of variance revealed that there were significant differences in growth rates at both 4 and 7 °C among CC types by OD_600_ measurement. Some differences and similarities were noted with the previous studies, e.g., CC14 isolates showed higher growth rates but CC4 isolates did not [7]. The discrepancies could be due to the different numbers of CC isolates tested. We have also shown that growth rates among the CC types at both temperatures were consistent with growth rates calculated using viable plate count data (Figure 5), e.g., CC18 exhibited higher growth and CC3, CC5 and CC121 isolates exhibited slower growth at each tested temperature. Overall the results highlight CC-types of particular concern, specifically CC18 and phylogenetically closely related CC types that grow faster in cold conditions and could pose particular problems if they contaminate food products relying on chill distribution for safety. It was noted that CC18, CC26 and CC8 types that caused listeriosis outbreaks in Switzerland between 2005–2011 [28,29] all grew faster than other CC types at low temperatures in this study. 

Another concern is whether the observed phenotype of CC18 is captured by the widely employed ComBase predictor. As seen in Figure 5, CC18 growth rates were statistically higher than the ComBase prediction line. However, the growth rates of all seven strains at the two different temperatures were within the 26th percentile of the ComBase prediction mean. Therefore, our findings are captured by the ComBase predictor (ComBase prediction allows 68 percentiles of uncertainty). Nonetheless, our study confirms that differences in growth rates in cold conditions are associated with CC type.

The tolerance of *L. monocytogenes* to specific stress conditions (e.g., pH, undissociated acid concentration, salt and low temperature) has been associated with the presence of particular stress response genes in the genome (Supplementary Table S1). To explore such associations, Scoary, pan-genome-wide association analysis and BLASTN approaches were employed to examine our collection of isolates. Although specific associations with faster growth rates at low temperatures were apparent for 15 out of 33 genes previously linked to cold tolerance by BLASTN analysis (see Figure 4), Scoary did not identify these genes. Instead Scoary selected six potential genes (with unknown function) associated with faster growth at low temperatures (Table 3). A similar study of the ability of *L. monocytogenes* to grow in BHIB at 2 °C and gene-based association analysis identified 114 genes that were associated with the ability to grow at 2 °C, but none were known stress tolerance genes, and 70 of the identified genes were only hypothetical [15]. However, it should be noted that previously reported cold tolerance genes were not identified in Prokka-annotated files in our study. The widely employed Prokka algorithm utilised multiple tools and stages to identify the coordinates of genomic features within WGS contigs but does not describe the putative gene product [20]. This limitation which is primarily due to Prokka’s functional annotation parameters, which are particularly stringent since Prokka annotates based on specific reference proteins with experimental evidence. Our study employed the orthologous genes database rather than *L. monocytogenes* EDG-e genes while annotating the DNA sequences, so as not to limit or bias the prediction of gene functions. Additionally, in Prokka, 48% of proteins were unannotated [30] and are thus classed as hypothetical proteins.

Evidence of variation in growth rates at low temperatures in broth in this and other studies raised the important question of whether these differences would also be observable in food matrices. Growth rate differences between laboratory media and complex food matrices have previously been demonstrated under stress conditions [31,32]. Therefore, to investigate strain variation effects on growth at low temperatures, commercially sterile milk, chocolate milk and fish pie were chosen (Table 1). Similar trends were observed, e.g., the CC18-type isolate demonstrated statistically faster growth than isolates of other CC types, specifically CC121, for milk and fish pie. It should be noted that direct comparison of growth rates between media and food matrices were not possible due to the variation of temperature in the standard equipment employed in the study. This did not impact the primary finding of this study: that cold tolerance is consistent with CC type in media and real food matrices. The CC18 isolates exhibited significantly faster growth in all the tested media and food matrices with the sole exception of chocolate milk where the higher growth rate was not statistically significant. It is worth noting that all of the growth rates in food matrices were below the ComBase prediction except fish pie with the CC18 strain. However, it was clear that specific CC types such as CC18 in food products or on the premises of food manufacturers may be more problematic for the industry.

## 5. Conclusions

This study used a large set of isolates (*n* = 150) to analyse the propensity of *L. monocytogenes* to grow at refrigeration temperatures. The majority of the isolates that grew faster at low temperatures were found to cluster by MLST and gene-based phylogeny methods. The cold tolerance trends observed for specific isolates in the laboratory media were also consistent in real food matrices. Collectively, we highlighted the usefulness and importance of typing strategies for the identification of stress tolerant variants of *L. monocytogenes*.

## Figures and Tables

**Table 1:** Details of test food matrices.

Product	pH	List of Ingredients
Milk (UHT)	6.77	UHT Milk 3.2% fat content
Chocolate milk (UHT)	6.89	Semi Skimmed Milk (96%), Sugar, Fat Reduced Cocoa Powder, Stabilisers (Guar Gum, Carrageenan), Flavourings, Vitamin D.
Fish pie	6.06	Organic Whole Milk 35%, Organic Carrots 19%, Organic Potatoes 19%, Organic Salmon (Fish) 10%, Organic Green Beans 7%, Organic Leeks 4%, Organic Onions 2%, Organic Breadcrumbs (Wheat Flour, Yeast) 2%, Organic Unsalted Butter (Milk) 1%, Organic Parsley 0.2%, Organic Peppercorns < 0.1%

**Table 2:** Isolates selected for detailed growth rate experiments in media and food matrices.

Isolates	CC	Category
LmF2365 *	1	Reference Isolate
Lm1372	3	Slow growth
Lm1378	5	Slow growth
Lm1991 *	121	Slow growth
Lm1515	18	Fast Growth
Lm3443 *	18	Fast Growth
Lm3448	18	Fast Growth

* These isolates were chosen for further experiments in food matrices.

**Table 3:** Scoary identified genes specific to cold tolerance and their prediction function (BLASTX).

Gene	Accession	Query (bp)	Identity (%)	Predicted Function
group_3291	WP_003727783.1	591	100	phage scaffolding protein
group_2594	WP_003734899.1	825	100	phage tail family protein
group_2595	HAC4747847.1	405	98.51	hypothetical protein
group_4413	EAG7277673.1	1014	99.7	phage tail protein
group_4415	EAF7046744.1	444	99.32	hypothetical protein
group_2328	WP_003727788.1	408	99.26	minor capsid protein

## Data Availability

Genome sequence data was previously published [6] and gorwth data will be available through Combase (www.combase.cc, accessed on 29 November 2022).

## Supplementary Materials

The following supporting information can be downloaded at: https://www.mdpi.com/article/10.3390/microorganisms11010065/s1, references [33,34,35,36,37,38,39,40,41,42,43,44,45] are cited in the Supplementary file.
