# Supplementary material for: Variability in Cold Tolerance of Food and Clinical Listeria monocytogenes Isolates
Source: Microorganisms. 2022 Dec 26;11(1):65. doi: 10.3390/microorganisms11010065 (PMC9862054; doi:10.3390/microorganisms11010065)
Supplement: Supplementary file 1 [file microorganisms-11-00065-s001.zip › microorganisms-2101181-supplementary.pdf]

Supplementary Table S1. List of genes previously shown to be linked to cold tolerance in *L. Monocytogenes*.

Gene name	Aliases	Function	Temperatur e	Assay/Remark	References
OppA	*lmo2569*	similar to dipeptide ABC transporter (dipeptide-binding protein)	5°C	A strain LO28 deletion mutant exhibited lower growth rate than wildtype (WT)	[1]
gbuA	*lmo1014*	highly similar to glycine betaine ABC transporter (ATP-binding protein)	4°C	Deletion mutant exhibited lower growth rate against WT using 10403S strain.	[2]
gbuB	*lmo1015*	highly similar to glycine betaine ABC transporters (permease)	4°C	Deletion mutant exhibited lower growth rate against WT using 10403S strain.	[2]
gbuC	*lmo1016*	highly similar to glycinebetaine ABC transporters (permease)	4°C	Deletion mutant exhibited lower growth rate against WT using 10403S strain.	[2]
betL	*lmo2092*	glycine betaine transporter	4°C	Deletion mutant exhibited lower generation time against WT using 10403S strain.	[2]
OpuCA	*lmo01428*	similar to glycine betaine/carnitine/choline ABC transporter (ATP-binding protein)	4°C	Deletion mutant exhibited lower generation time against WT using 10403S strain.	[2]
OpuCB	*lmo01427*	similar to glycine betaine/carnitine/choline ABC transporter (membrane protein)	4°C	Deletion mutant exhibited lower generation time against WT using 10403S strain.	[2]

OpuCC	*lmo01426*	similar to glycine betaine/carnitine/choline ABC transporter (osmoprotectant-binding protein)	4°C	Deletion mutant exhibited lower generation time against WT using 10403S strain.	[2]
OpuCD	*lmo01425*	similar to betaine/carnitine/choline ABC transporter (membrane p)	4°C	Deletion mutant exhibited lower generation time against WT using 10403S strain.	[2]
flaA	*lmo00690*	flagellin protein	10°C	The genes was expressed in response to growth at low temperature	[3]
fbP	*lmo00830*	highly similar to fructose-1,6-bisphosphatase	10°C	Genes expressed in response to growth at low temperature	[3]
orfX	*lmo0206*	hypothetical protein	3°C	Deletion mutant exhibited longer lag time then WT using EGD strain	[4]
sigB	*lmo00895*	RNA polymerase sigma-37 factor (sigma-B)	8°C	Deletion mutant shown double lag time compared to WT using 10403S starin	[5]
rpoN (sigL)	*lmo02461*	RNA polymerase sigma-54 factor (sigma-L)	10°C	Genes Expressed in Response to Growth at Low Temperature	[3]
bglG	*lmo00501*	similar to transcription antiterminator BglG family	10°C	Genes Expressed in Response to Growth at Low Temperature	[3]

hfq	*lmo01295*	similar to host factor-1 protein	4°C	Mutant strain displayed a slightly prolonged lag phase than WT using EGD strain.	[6]
degU	*lmo02515*	similar to B. subtilis two-component response regulator	25°C	Transcriptional activator of flaA gene, in EGD strain	[7]
yycJ	*lmo00291*	conserved hypothetical protein similar to *B. subtilis YycJ* protein	10°C	Genes expressed in response to growth at low temperature in 10403S strain	[3]
lhkA	*lmo1508*	similar to two-component sensor histidine kinase	10°C	Genes expressed in response to growth at low temperature in 10403S strain	[3]
psr	*lmo0443*	similar to *B. subtilis* transcription regulator *LytR*	10°C	Genes expressed in response to growth at low temperature in 10403S strain	[3]
cspL, cspA	*lmo01364*	similar to cold shock protein	4, and 10°C	Gene expressed in response to cold shock at 10 °C in LO28 strain	[8]
cspD	*lmo01879*	similar to major cold-shock protein	4, and 10°C	Deletion mutant exhibited longer lag time then WT using EGDe strain	[8]
fri (flp)	*lmo0943*	non-heme iron-binding ferritin	10°C	Genes expressed in response to grow at 10°C	[3]
trxB	*lmo2478*	thioredoxin reductase	10°C	Genes expressed in response to grow at 10°C	[3]

aroA	*lmo1600*	3-deoxy-D-arabino-heptulosonate 7-phosphate synthase	10°C	Genes expressed in response to grow at 10°C	[3]
cysS	*lmo0239*	Cysteinyl-tRNA synthetase	10°C	Genes expressed in response to grow at 10°C	[3]
trpG	*lmo1632*	highly similar to anthranilate synthase beta subunit	10°C	Genes expressed in response to grow at 10°C	[3]
ltrA	*lmo0389*	low temperature requirement protein A	4°C	Genes Expressed in Response to Growth at Low Temperature	[9]
ltrB	*lmo0215*	low temperature requirement B protein	4°C	Genes Expressed in Response to Growth at Low Temperature	[9]
ltrC	*lom2398*	low temperature requirement C protein	4°C	Genes Expressed in Response to Growth at Low Temperature	[9]
groEL	*lmo2068*	class I heat-shock protein (chaperonin) *GroEL*	10°C	Multiple strains Genes Expressed in Response to Growth at Low Temperature	[3]
clpP	*lmo2468*	ATP-dependent *Clp* protease proteolytic subunit	10°C	Genes Expressed in Response to Growth at Low Temperature	[3]
clpB	*lmo2206*	similar to endopeptidase *Clp* ATP-binding chain B (*ClpB*)	10°C	Genes Expressed in Response to Growth at Low Temperature	[3]

deaD	*lmo0866*	similar to ATP-dependent RNA helicase	3°C	Deletion mutant Vs wildtype EGDe No difference growth rate but shorten lag in WT	[10]
deaD	*lmo1450*	similar to ATP-dependent RNA helicase, DEAD-box family (*deaD*)	3°C	Deletion mutant Vs wildtype EGDe No difference growth rate but shorten lag in WT	[10]
deaD	*lmo1722*	similar to ATP-dependent RNA helicase, DEAD-box family (*deaD*)	3°C	Deletion mutant Vs wildtype EGDe No difference growth rate but shorten lag in WT	[10]
pgpH	*lmo1466*	similar to unknown proteins	3°C	Genes Expressed in Response to Growth at Low Temperature	[11]
yycG	*lmo0288*	Sensors (signal transduction)	3°C	Genes Expressed in Response to Growth at Low Temperature	[12]
LisR	*lmo01377*	two-component response regulator	3°C	Genes Expressed in Response to Growth at Low Temperature	[12]
resE	*lmo01947*	similar to two-component sensor histidine kinase (*ResE*)	3°C	Deletion mutant Vs wildtype EGDe low growth rate than WT	[12]
flhA	*lmo0680*	similar to flagella-associated protein *flhA*	3°C	Deletion mutant Vs wildtype EGDe low growth rate than WT	[13]

MotA	*lmo0685*	similar to motility protein (flagellar motor rotation) *MotA*	3°C	Deletion mutant Vs wildtype EGDe low growth rate than WT	[13]

Supplementary Table S2. Isolate characteristics and phenotypic traits.

ID	Source	CC	Lineage	Serogroup	Plasmid	µ_4C	µ_7C	H_Cluster
Lm966	Environment	CC220	l	4b,4d,4e	Absent	0.0123	0.0179	Slow
Lm974	Environment	CC121	ll	1/2a,3a	Present	0.0134	0.0153	Slow
Lm976	Mixed food	CC20	ll	1/2a,3a	Absent	0.0138	0.0241	Fast
Lm1055	Environment	CC59	l	1/2b,3b,7	Absent	0.0110	0.0127	Slow
Lm1093	Environment	CC59	l	1/2b,3b,7	Absent	0.0128	0.0176	Slow
Lm1095	Environment	CC37	ll	1/2a,3a	Absent	0.0149	0.0264	Fast
Lm1123	Seafood	CC121	ll	1/2a,3a	Present	0.0138	0.0227	Fast
Lm1203	Environment	CC18	ll	1/2a,3a	Absent	0.0148	0.0292	Fast
Lm1268	Dairy	CC224	l	1/2b,3b,7	Absent	0.0131	0.0125	Slow
Lm1304	Environment	NOVEL	ll	1/2a,3a	Present	0.0155	0.0212	Fast
Lm1306	Environment	NOVEL	ll	1/2a,3a	Present	0.0146	0.0243	Fast
Lm1309	Environment	CC204	ll	1/2a,3a	Present	0.0144	0.0207	Fast
Lm1313	Environment	CC121	ll	1/2a,3a	Present	0.0144	0.0227	Fast
Lm1321	Environment	CC101	ll	1/2a,3a	Present	0.0118	0.0200	Slow
Lm1370	Meat	CC9	ll	1/2c,3c	Absent	0.0155	0.0284	Fast
Lm1371	Meat	CC31	ll	1/2a,3a	Present	0.0118	0.0138	Slow
Lm1372	Meat	CC3	l	1/2b,3b,7	Present	0.0140	0.0143	Slow
Lm1373	Meat	CC121	ll	1/2a,3a	Present	0.0128	0.0172	Slow
Lm1374	Meat	CC3	l	1/2b,3b,7	Present	0.0140	0.0120	Slow
Lm1375	Meat	CC2	l	4b,4d,4e	Absent	0.0127	0.0178	Slow
Lm1376	Meat	CC3	l	1/2b,3b,7	Absent	0.0131	0.0161	Slow
Lm1377	Meat	CC2	l	4b,4d,4e	Absent	0.0130	0.0165	Slow
Lm1378	Dairy	CC5	l	1/2b,3b,7	Present	0.0136	0.0175	Slow
Lm1379	Dairy	CC14	ll	1/2a,3a	Absent	0.0146	0.0272	Fast
Lm1380	Meat	CC2	l	4b,4d,4e	Absent	0.0129	0.0178	Slow
Lm1381	Meat	CC2	l	4b,4d,4e	Absent	0.0112	0.0159	Slow
Lm1382	Dairy	CC4	l	4b,4d,4e	Absent	0.0142	0.0153	Slow
Lm1383	Dairy	CC412	ll	1/2a,3a	Absent	0.0148	0.0212	Fast
Lm1384	Seafood	CC59	l	1/2b,3b,7	Absent	0.0112	0.0166	Slow
Lm1385	Seafood	CC155	ll	1/2a,3a	Absent	0.0150	0.0259	Fast
Lm1386	Seafood	CC5	l	1/2b,3b,7	Present	0.0135	0.0185	Slow
Lm1387	Seafood	CC2	l	4b,4d,4e	Absent	0.0157	0.0171	Fast
Lm1388	Seafood	CC2	l	4b,4d,4e	Absent	0.0130	0.0183	Slow
Lm1389	Seafood	CC31	ll	1/2a,3a	Present	0.0151	0.0168	Fast
Lm1390	Dairy	CC4	l	4b,4d,4e	Absent	0.0132	0.0133	Slow
Lm1391	Dairy	CC54	l	4b,4d,4e	Absent	0.0115	0.0125	Slow
Lm1392	Seafood	CC6	l	4b,4d,4e	Absent	0.0139	0.0134	Slow
Lm1393	Meat	CC2	l	4b,4d,4e	Absent	0.0142	0.0167	Slow
Lm1394	Dairy	CC26	ll	1/2a,3a	Absent	0.0178	0.0224	Fast
Lm1403	Seafood	CC121	ll	1/2a,3a	Present	0.0156	0.0140	Fast
Lm1411	Environment	CC1	l	4b,4d,4e	Absent	0.0127	0.0137	Slow
Lm1413	Environment	CC2	l	4b,4d,4e	Absent	0.0127	0.0117	Slow
Lm1423	Environment	CC121	ll	1/2a,3a	Present	0.0129	0.0100	Slow
Lm1427	Environment	NOVEL	ll	1/2a,3a	Present	0.0152	0.0179	Fast
Lm1428	Environment	NOVEL	ll	1/2a,3a	Present	0.0162	0.0201	Fast
Lm1439	Seafood	CC121	ll	1/2a,3a	Present	0.0125	0.0125	Slow
Lm1441	Environment	CC5	l	1/2b,3b,7	Present	0.0117	0.0127	Slow

Lm1445	Meat	CC7	ll	1/2a,3a	Absent	0.0121	0.0135	Slow
Lm1502	Environment	NOVEL	ll	1/2a,3a	Absent	0.0160	0.0189	Fast
Lm1507	Environment	NOVEL	ll	1/2a,3a	Absent	0.0141	0.0192	Fast
Lm1513	Seafood	CC121	ll	1/2a,3a	Present	0.0137	0.0162	Slow
Lm1515	Environment	CC18	ll	1/2a,3a	Absent	0.0155	0.0210	Fast
Lm1519	Seafood	CC121	ll	1/2a,3a	Present	0.0126	0.0181	Slow
Lm1527	Environment	CC37	ll	1/2a,3a	Absent	0.0148	0.0226	Fast
Lm1534	Meat	CC6	l	4b,4d,4e	Absent	0.0121	0.0162	Slow
Lm1564	Environment	CC2	l	4b,4d,4e	Absent	0.0132	0.0150	Slow
Lm1679	Environment	CC204	ll	1/2a,3a	Absent	0.0163	0.0183	Fast
Lm1798	Dairy	CC18	ll	1/2a,3a	Absent	0.0152	0.0207	Fast
Lm1880	Vegetable	CC8	ll	1/2a,3a	Present	0.0137	0.0222	Fast
Lm1976	Environment	CC8	ll	1/2a,3a	Present	0.0147	0.0275	Fast
Lm1989	Seafood	CC121	ll	1/2a,3a	Present	0.0126	0.0197	Slow
Lm1991	Seafood	CC121	ll	1/2a,3a	Present	0.0125	0.0168	Slow
Lm2075	Environment	CC14	ll	1/2a,3a	Absent	0.0148	0.0254	Fast
Lm2095	Environment	CC54	l	4b,4d,4e	Absent	0.0057	0.0107	Slow
Lm2105	Environment	CC54	l	4b,4d,4e	Absent	0.0120	0.0128	Slow
Lm2113	Environment	CC1	l	4b,4d,4e	Absent	0.0123	0.0102	Slow
Lm2181	Environment	CC220	l	4b,4d,4e	Absent	0.0113	0.0127	Slow
Lm2183	Environment	CC2	l	4b,4d,4e	Absent	0.0153	0.0155	Fast
Lm2185	Environment	CC14	ll	1/2a,3a	Absent	0.0165	0.0194	Fast
Lm2226	Environment	CC220	l	4b,4d,4e	Absent	0.0166	0.0139	Fast
Lm2234	Environment	CC220	l	4b,4d,4e	Absent	0.0147	0.0145	Fast
Lm2237	Environment	CC2	l	4b,4d,4e	Absent	0.0079	0.0185	Slow
Lm2240	Environment	CC59	l	1/2b,3b,7	Absent	0.0134	0.0127	Slow
Lm2241	Environment	CC59	l	1/2b,3b,7	Absent	0.0143	0.0150	Slow
Lm2256	Environment	CC121	ll	1/2a,3a	Absent	0.0192	0.0258	Fast
Lm2259	Environment	CC54	l	4b,4d,4e	Absent	0.0125	0.0166	Slow
Lm2294	Environment	CC8	ll	1/2a,3a	Present	0.0153	0.0217	Fast
Lm2298	Environment	CC379	l	1/2b,3b,7	Absent	0.0132	0.0153	Slow
Lm2507	Environment	CC220	l	4b,4d,4e	Absent	0.0133	0.0135	Slow
Lm3442	Vegetable	CC87	l	1/2b,3b,7	Absent	0.0114	0.0134	Slow
Lm3443	Vegetable	CC18	ll	1/2a,3a	Absent	0.0174	0.0196	Fast
Lm3444	Vegetable	CC1	l	4b,4d,4e	Absent	0.0134	0.0124	Slow
Lm3445	Vegetable	CC54	l	4b,4d,4e	Absent	0.0124	0.0129	Slow
Lm3446	Vegetable	CC54	l	4b,4d,4e	Absent	0.0129	0.0168	Slow
Lm3447	Vegetable	CC59	l	1/2b,3b,7	Absent	0.0139	0.0130	Slow
Lm3448	Vegetable	CC18	ll	1/2a,3a	Absent	0.0183	0.0212	Fast
Lm6179	Dairy	CC121	ll	1/2c,3c	Present	0.0165	0.0174	Fast
Lm10403S	Clinical	CC7	ll	1/2a,3a	Absent	0.0148	0.0171	Fast
Lm1513C	Environment	NOVEL	ll	1/2a,3a	Absent	0.0120	0.0197	Slow
LmEGD-e	Clinical	CC9	ll	1/2c,3c	Absent	0.0168	0.0199	Fast
LmF1109-17	Environment	CC121	ll	1/2a,3a	Present	0.0084	0.0105	Slow
LmF111-17	Mixed food	CC121	ll	1/2a,3a	Present	0.0140	0.0156	Slow
LmF112-17	Mixed food	CC121	ll	1/2a,3a	Present	0.0137	0.0141	Slow
LmF114-17	Mixed food	CC121	ll	1/2a,3a	Present	0.0157	0.0174	Fast
LmF1209-16	Environment	CC121	ll	1/2a,3a	Present	0.0133	0.0141	Slow
LmF1221-16	Meat	CC121	ll	1/2a,3a	Present	0.0123	0.0150	Slow
LmF1230-16	Meat	CC121	ll	1/2a,3a	Present	0.0139	0.0120	Slow

LmF1524-16	Environment	CC5	l	1/2b,3b,7	Present	0.0130	0.0102	Slow
LmF1644-17	Environment	CC5	l	1/2b,3b,7	Present	0.0106	0.0152	Slow
LmF1646-17	Environment	CC121	ll	1/2a,3a	Present	0.0134	0.0147	Slow
LmF168-17	Environment	CC121	ll	1/2a,3a	Present	0.0131	0.0118	Slow
LmF1857-15	Environment	CC5	l	1/2b,3b,7	Present	0.0117	0.0134	Slow
LmF1994-15	Meat	CC220	l	4b,4d,4e	Absent	0.0119	0.0132	Slow
LmF2151-17	Meat	CC121	ll	1/2a,3a	Present	0.0125	0.0133	Slow
LmF2152-17	Meat	CC121	ll	1/2a,3a	Present	0.0127	0.0105	Slow
LmF2153-17	Meat	CC121	ll	1/2a,3a	Present	0.0125	0.0123	Slow
LmF2154-17	Meat	CC121	ll	1/2a,3a	Present	0.0121	0.0192	Slow
LmF2155-17	Meat	CC121	ll	1/2a,3a	Present	0.0124	0.0125	Slow
LmF2160-17	Meat	CC121	ll	1/2a,3a	Present	0.0125	0.0146	Slow
LmF2161-17	Environment	CC121	ll	1/2a,3a	Absent	0.0132	0.0125	Slow
LmF2165-17	Environment	CC121	ll	1/2a,3a	Present	0.0081	0.0113	Slow
LmF2166-17	Environment	CC5	l	1/2b,3b,7	Present	0.0100	0.0137	Slow
LmF2170-17	Environment	CC121	ll	1/2a,3a	Present	0.0080	0.0213	Slow
LmF2176-17	Environment	CC5	l	1/2b,3b,7	Present	0.0108	0.0156	Slow
LmF2299-15	Environment	CC5	l	1/2b,3b,7	Present	0.0095	0.0135	Slow
LmF2365	Dairy	CC1	l	4b,4d,4e	Absent	0.0122	0.0132	Slow
LmF340-17	Environment	CC121	ll	1/2a,3a	Present	0.0118	0.0146	Slow
LmF345-17	Environment	CC121	ll	1/2a,3a	Present	0.0117	0.0136	Slow
LmF347-16	Environment	CC5	l	1/2b,3b,7	Present	0.0173	0.0165	Fast
LmF347-17	Environment	CC121	ll	1/2a,3a	Present	0.0115	0.0146	Slow
LmF348-17	Environment	CC121	ll	1/2a,3a	Present	0.0111	0.0120	Slow
LmF365-16	Meat	CC121	ll	1/2a,3a	Present	0.0117	0.0146	Slow
LmF671-16	Environment	CC5	l	1/2b,3b,7	Present	0.0100	0.0149	Slow
LmF742-17	Environment	CC5	l	1/2b,3b,7	Present	0.0110	0.0134	Slow
LmF991-16	Environment	CC5	l	1/2b,3b,7	Present	0.0136	0.0136	Slow
LmMQ130026	Clinical	CC1	l	4b	Absent	0.0125	0.0151	Slow
LmMQ130029	Clinical	CC1	l	4b	Absent	0.0123	0.0151	Slow
LmMQ130032	Clinical	CC1	l	4b	Absent	0.0148	0.0160	Fast
LmMQ130033	Clinical	CC54	l	4b	Absent	0.0159	0.0179	Fast
LmMQ130037	Clinical	CC18	ll	1/2a	Present	0.0148	0.0237	Fast
LmMQ130042	Clinical	CC1	l	4b	Absent	0.0118	0.0156	Slow
LmMQ130058	Clinical	CC6	l	4b	Absent	0.0139	0.0136	Slow
LmMQ140011	Clinical	CC101	ll	1/2a	Absent	0.0141	0.0272	Fast
LmMQ140012	Clinical	CC101	ll	1/2a	Absent	0.0143	0.0261	Fast
LmMQ140025	Clinical	CC1	l	4b	Absent	0.0134	0.0206	Slow
LmMQ140029	Clinical	CC7	ll	1/2a	Absent	0.0142	0.0211	Fast
LmMQ140030	Clinical	CC4	l	4b	Absent	0.0081	0.0171	Slow
LmMQ140031	Clinical	CC1	l	4b	Absent	0.0104	0.0162	Slow
LmMQ140032	Clinical	CC90	ll	1/2a	Absent	0.0140	0.0237	Fast
LmMQ140033	Clinical	CC1	l	4b	Absent	0.0144	0.0269	Fast
LmMQ140034	Clinical	CC121	ll	1/2a	Absent	0.0139	0.0206	Fast
LmMQ140035	Clinical	CC121	ll	1/2a	Absent	0.0079	0.0184	Slow
LmMQ150001	Clinical	CC37	ll	1/2a	Absent	0.0146	0.0235	Fast
LmMQ150004	Clinical	CC54	l	4b	Absent	0.0123	0.0159	Slow
LmMQ150005	Clinical	CC6	l	4b	Absent	0.0123	0.0145	Slow
LmMQ150007	Clinical	CC101	ll	1/2a	Absent	0.0125	0.0202	Slow
LmMQ150008	Clinical	CC101	ll	1/2a	Present	0.0119	0.0205	Slow

LmMQ150011	Clinical	CC20	ll	1/2a	Present	0.0129	0.0211	Slow
LmMQ150012	Clinical	CC6	l	4b	Absent	0.0119	0.0157	Slow
LmMQ150013	Clinical	CC2	l	4b	Absent	0.0140	0.0164	Slow

Supplementary Figure S1. Rapid Alert System for Food and Feed (RASFF) food notifications in the EU for *L. Monocytogenes* from September 2020-March 2022 (available at https://webgate.ec.europa.eu/rasff-window/screen/search).

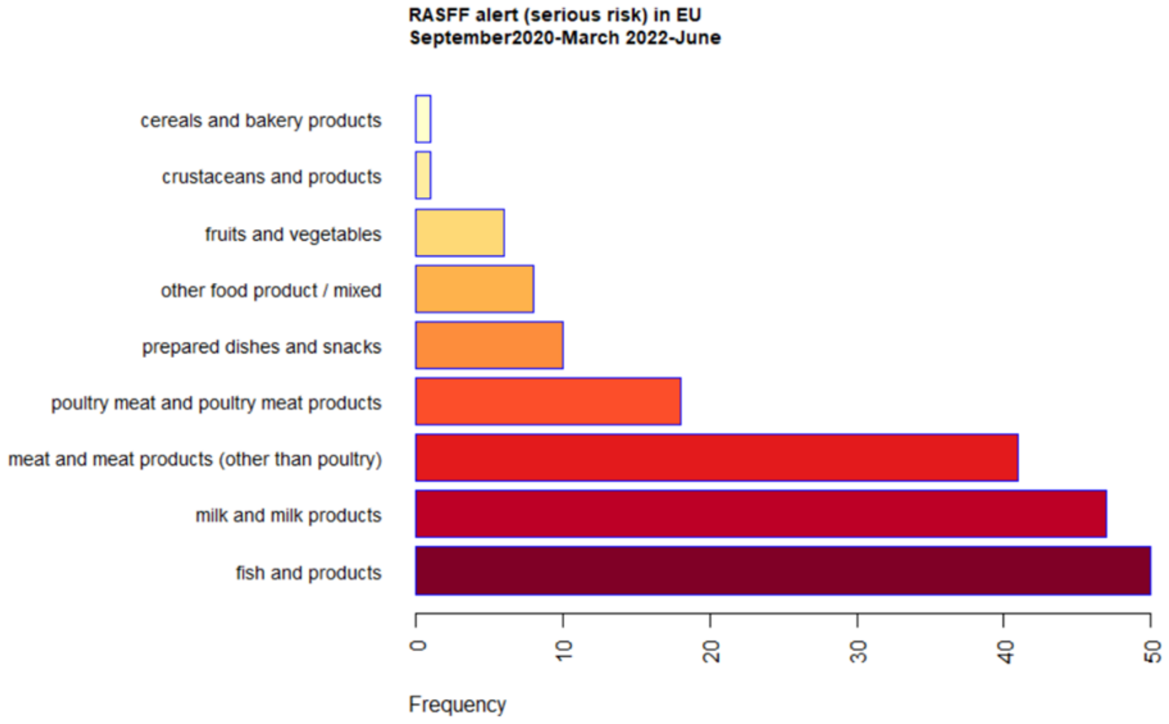

References

[1] Borezee, E.; Pellegrini, E.; Berche, P. OppA of *Listeria Monocytogenes*, an Oligopeptide-Binding Protein Required for Bacterial Growth at Low Temperature and Involved in Intracellular Survival. *Infect. Immun.*, **2000**, *68* (12), 7069–7077. https://doi.org/10.1128/IAI.68.12.7069-7077.2000.

[2] Angelidis, A. S.; Smith, G. M. Role of the Glycine Betaine and Carnitine Transporters in Adaptation of *Listeria Monocytogenes* to Chill Stress in Defined Medium. *Appl. Environ. Microbiol.*, **2003**, *69* (12), 7492–7498. https://doi.org/10.1128/AEM.69.12.7492-7498.2003.

[3] Liu, S.; Graham, J. E.; Bigelow, L.; Morse, P. D.; Wilkinson, B. J. Identification of *Listeria Monocytogenes* Genes Expressed in Response to Growth at Low Temperature. *Appl. Environ. Microbiol.*, **2002**, *68* (4), 1697–1705. https://doi.org/10.1128/AEM.68.4.1697-1705.2002.

[4] Brøndsted, L.; Kallipolitis, B. H.; Ingmer, H.; Knöchel, S. KdpE and a Putative RsbQ Homologue Contribute to Growth of *Listeria Monocytogenes* at High Osmolarity and Low Temperature. *FEMS Microbiol. Lett.*, **2003**, *219* (2), 233–239. https://doi.org/10.1016/S0378-1097(03)00052-1.

[5] Becker, L. A.; Evans, S. N.; Hutkins, R. W.; Benson, A. K. Role of ΣB in Adaptation of *Listeria Monocytogenes* to Growth at Low Temperature. *J. Bacteriol.*, **2000**, *182* (24), 7083–7087. https://doi.org/10.1128/JB.182.24.7083-7087.2000.

[6] Christiansen, J. K.; Larsen, M. H.; Ingmer, H.; Søgaard-Andersen, L.; Kallipolitis, B. H. The RNA-Binding Protein Hfq of *Listeria Monocytogenes*: Role in Stress Tolerance and Virulence. *J. Bacteriol.*, **2004**, *186* (11), 3355–3362. https://doi.org/10.1128/JB.186.11.3355-3362.2004.

[7] Knudsen, G. M.; Olsen, J. E.; Dons, L. Characterization of DegU, a Response Regulator in *Listeria Monocytogenes*, Involved in Regulation of Motility and Contributes to Virulence. *FEMS Microbiol. Lett.*, **2004**, *240* (2), 171–179. https://doi.org/10.1016/j.femsle.2004.09.039.

[8] Schmid, B.; Klumpp, J.; Raimann, E.; Loessner, M. J.; Stephan, R.; Tasara, T. Role of Cold Shock Proteins in Growth of *Listeria Monocytogenes* under Cold and Osmotic Stress Conditions. *Appl. Environ. Microbiol.*, **2009**, *75* (6), 1621–1627. https://doi.org/10.1128/AEM.02154-08.

[9] Zheng, W.; Kathariou, S. Differentiation of Epidemic-Associated Strains of *Listeria Monocytogenes* by Restriction Fragment Length Polymorphism in a Gene Region Essential for Growth at Low Temperatures (4°C). *Appl. Environ. Microbiol.*, **1995**, *61* (12), 4310–4314. https://doi.org/10.1128/aem.61.12.4310-4314.1995.

[10] Markkula, A.; Mattila, M.; Lindström, M.; Korkeala, H. Genes Encoding Putative DEAD-Box RNA Helicases in *Listeria Monocytogenes* EGD-e Are Needed for Growth and Motility at 3°C. *Environ. Microbiol.*, **2012**, *14* (8), 2223–2232. https://doi.org/10.1111/j.1462-2920.2012.02761.x.

[11] Arguedas-Villa, C.; Stephan, R.; Tasara, T. Evaluation of Cold Growth and Related Gene Transcription Responses Associated with *Listeria Monocytogenes* Strains of Different Origins. *Food Microbiol.*, **2010**, *27* (5), 653–660. https://doi.org/10.1016/j.fm.2010.02.009.

[12] Pöntinen, A.; Markkula, A.; Lindström, M.; Korkeala, H. Two-Component-System Histidine Kinases Involved in Growth of *Listeria Monocytogenes* EGD-e at Low Temperatures. *Appl. Environ. Microbiol.*, **2015**, *81* (12), 3994–4004. https://doi.org/10.1128/AEM.00626-15.

[13] Mattila, M.; Lindström, M.; Somervuo, P.; Markkula, A.; Korkeala, H. Role of FlhA and MotA in Growth of *Listeria Monocytogenes* at Low Temperatures. *Int. J. Food Microbiol.*, **2011**, *148* (3), 177–183. https://doi.org/10.1016/j.ijfoodmicro.2011.05.022.
